# Cellulose Nanocrystals vs. Cellulose Nanofibers: A Comparative Study of Reinforcing Effects in UV-Cured Vegetable Oil Nanocomposites

**DOI:** 10.3390/nano11071791

**Published:** 2021-07-09

**Authors:** Anda Barkane, Edgars Kampe, Oskars Platnieks, Sergejs Gaidukovs

**Affiliations:** Institute of Polymer Materials, Faculty of Materials Science and Applied Chemistry, Riga Technical University, P. Valdena 3/7, LV-1048 Riga, Latvia; Anda.Barkane@rtu.lv (A.B.); Edgars.Kampe@rtu.lv (E.K.); Oskars.Platnieks_1@rtu.lv (O.P.)

**Keywords:** biobased polymer, nanocellulose, UV-curing, green renewable materials, photopolymerization, acrylated epoxidized soybean oil, thermomechanical properties, thermal properties

## Abstract

There is an opportunity to use nanocellulose as an efficient renewable reinforcing filler for polymer composites. There have been many investigations to prove the reinforcement concept of different nanocellulose sources for thermoplastic and thermoset polymers. The present comparative study highlighted the beneficial effects of selecting cellulose nanofibers (CNFs) and nanocrystals (CNCs) on the exploitation properties of vegetable oil-based thermoset composite materials—thermal, thermomechanical, and structural characteristics. The proposed UV-light-curable resin consists of an acrylated epoxidized soybean oil polymer matrix and two different nanocellulose reinforcements. High loadings of up to 30 wt% of CNFs and CNCs in irradiation-cured vegetable oil-based thermoset composites were reported. Infrared spectroscopy analysis indicated developed hydrogen-bonding interactions between the nanocellulose and polymer matrix. CNCs yielded a homogeneous nanocrystal dispersion, while CNFs revealed a nanofiber agglomeration in the polymer matrix, as shown by scanning electron microscopy. Thermal degradation showed that nanocellulose reduced the maximum degradation temperature by 5 °C for the 30 wt% CNC and CNF nanocomposites. Above the glass transition temperature at 80 °C, the storage modulus values increased 6-fold and 2-fold for the 30 wt% CNC and CNF nanocomposites, respectively. In addition, the achieved reinforcement efficiency factor r value for CNCs was 8.7, which was significantly higher than that of CNFs of 2.2. The obtained nanocomposites with enhanced properties show great potential for applications such as UV-light-processed coatings, adhesives, and additive manufacturing inks.

## 1. Introduction

Annual global plastic and rubber production is approaching 400 million tons, a significant increase from just a few million tons 50 years ago [1]. Although polymers are versatile materials, the rapid growth has resulted in an industry built around fossil feedstock and their applications [2], making this dependence unsustainable in the long term. On the other hand, the extensive use of fossil resources has resulted in a substantial environmental impact through greenhouse gas emissions. It has become urgent to develop sustainable, green, and renewable technologies toward high-performance materials to replace conventional plastics and to alleviate these problems. Indeed, vegetable oils have proven to be a suitable raw material for thermoset polymer resin. Vegetable oils meet the potential demand as they are available in large quantities from various crops in all climate conditions [3,4,5]. Nanocellulose is one of the most prospective green nanomaterials owing to its versatility, abundant renewable sources, and highly developed processing technologies [6,7]. Some of the critical characteristics of nanocellulose are mechanical strength, high elastic modulus, adjustable surface chemistry, barrier properties, and nontoxicity [8]. Combining these renewable materials combines some of the finest properties that can yield highly functional advanced materials for applications in additive manufacturing, surface coating, and the preparation of bio-based adhesives.

The double bonds in fatty acids of vegetable oils can polymerize through oxidation, but still, such a process is slow and often leads to an inconsistent quality of the produced materials [3]. Thus, converting double bonds to epoxy, hydroxyl, thiol, and other active functional groups in oils rich in unsaturated fatty acid moieties such as oleic, linoleic, and linolenic presents an attractive path for the conversion of vegetable oils to polymeric materials [4,9]. Among these oil derivatives, epoxidized vegetable oils have been used in many polymerization processes, particularly in photo-initiated polymerization, yet with only partial success due to their high viscosity, resulting in low layer formation and low-performance characteristics [10,11]. Alternatively, the introduction of acrylate moieties to epoxidized acrylate oils reduces the viscosity, increases thermal stability, and adds higher reactivity to the resin formulation, as reported previously by authors [12]. While toxicity issues have been brought up regarding the use of acrylates, it is known that monomer and oligomer properties do not transfer over to the polymer, which is usually chemically stable, nonreactive, and nonhazardous [13]. Unfortunately, polymeric epoxidized acrylates obtained from vegetable oils compared to fossil-based resins still show relatively low mechanical properties. Therefore, a common practice is to enhance bio-based resins with polyfunctional synthetic reactive diluents to control radical polymerization chain growth and branching [14,15].

Several studies have investigated the use of bio-based reactive diluents for vegetable oil-based resins, such as cardanyl acrylate [16], acrylated betulin [17], glycidyl methacrylate [18], and tung oil-based methacrylate [11]. In addition to bio-based reactive diluents that are still not enough to achieve high performances in terms of mechanical performance, many studies have demonstrated the high potential of using bio-based fillers to enhance the mechanical characteristics of the materials while complying with the green concept of advanced biocomposites [19,20]. Nanofillers such as different types of nanocellulose can preserve good photoinitiator activity in the resin formulation and a high precision of selected coating techniques or the stereolithography (SLA) setup.

Cellulose nanocrystals (CNCs) are preferred for their chemical purity, crystallinity, mechanical strength, and optical characteristics, while their drawbacks include extensive chemical treatment and leftover acidic water in the production process that needs additional purification [21]. Cellulose nanofibers (CNFs) reduce the need for aggressive chemical treatment, often including enzymatic or chemical pretreatments, but the primary production step consists of mechanical delamination achieved with high-pressure homogenizers or ultra-fine grinders, resulting in a network of cellulose fibrils with high surface area and high specific aspect ratio [21,22]. Xu et al. demonstrated a comparison of both fillers in the polymer matrix with loadings up to 10 wt%, showing that CNFs can achieve a higher strength and modulus but tend to agglomerate, resulting in a lower strain-at-failure [23]. Thus, various factors such as compatibility with matrix and its original properties must be considered for the best filler selection, while issues with CNF agglomeration have led to CNCs being preferred for high-performance applications [24,25].

Epoxidized sunflower oil/CNC composites increased the mechanical properties compared to the polymer matrix, but exposure to water significantly influenced the performance; thus, the authors suggested sensory application [26]. Acrylated epoxidized soybean oil (AESO)/CNC composites with loadings up to 2 wt% were reported to increase the hardness Gouge resistance from F to 6H, scratch resistance from B to H, and overall tensile properties, which revealed a higher increase with modified CNCs [27]. It was shown that 0.1 wt% of CNF filler increased the epoxy resin composite’s Young’s modulus two times and the toughness almost five times [28]. Wang et al. demonstrated a methacrylic acid resin composite for SLA applications with CNCs, tetracarboxylic butane acid, and sodium hypophosphite fillers, and CNCs as a single filler for enhanced mechanical, thermal, and dynamic mechanical properties [29]. Methacrylic-siloxane-microcrystalline cellulose composite coatings for wood protection were applied on walnut wood samples; thus, hydrophobicity was lowered and a decreased thermal expansion coefficient proportional to the filler content (5 and 10 wt%) was observed [30]. Cataldi et al. reported that photocurable resin loaded with nanocellulose resulted in an increased glass transition temperature, thermal and dimensional stability, and stiffness of the nanocomposite [19]. In addition, CNCs increased the water uptake, i.e., hydrophilic surface properties for some composites. CNCs have also been reported to work as an effective barrier for water vapors [31], making them suitable for wood surface coating applications [30]. Construction, engineering, rapid prototyping, sensors, fibers, and coatings greatly benefit from introducing nanocellulose-based fillers into the polymer matrix [32,33].

The CNFs and CNCs as reinforcing materials have gained significant interest for nanocomposites. However, studies that focus on comparing these two structurally very different types of nanocellulose in vegetable oil-based UV-cured thermosets are lacking. The present study aimed to compare the effects of CNCs and CNFs on the structural, thermomechanical, thermal stability, and photoinduced curing properties of UV-curable acrylated epoxidized soybean oil resin. The proposed broad content of nanocellulose from 5 up to 30 wt% expands the understanding of the reinforcing potential of CNCs (rod-like nanoparticles) and CNFs (flexible nanofibers) in the nanocomposites. CNCs and CNFs have been assessed for renewable nanocomposite formulation, and photocuring has been discussed considering their interactions with the polymer matrix. The article is written as a comprehensive comparison between nanocellulose fillers.

## 2. Materials and Methods

### 2.1. Materials

Acrylated epoxidized soybean oil (AESO) (contains 3500–4500 ppm monomethyl ether hydroquinone as an inhibitor, viscosity 18,000–32,000 cps.), reactive diluents trimethylolpropane triacrylate (TMPTA) (purity of >70.00%, contains 500–750 ppm monomethyl ether hydroquinone as inhibitor) and 1,6-hexanediol diacrylate-technical grade (HDDA) (purity 77.5%), and photoinitiator (PI) diphenyl(2,4,6-trimethyl-benzoyl)phosphine oxide (TPO) were used. All chemicals were purchased from Merck KGaA (Darmstadt, Germany) and used as received. Cellulose nanocrystals (CNCs) and cellulose nanofibers (CNFs) were obtained from Stucken Melchers GmbH and Co. (Bremen, Germany) and FCBA (Champs-sur-Marne, France), correspondingly. Both nanocellulose water dispersions were kindly provided by the Luxembourg Institute of Science and Technology (LIST) and used without additional manipulations.

**Figure 1:**
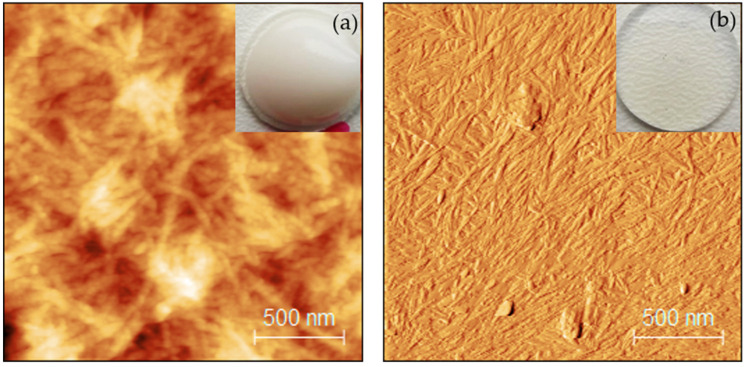
AFM images of CNFs (**a**) and CNCs (**b**) with water solution insets in the top right corners.

Dynamic light scattering (DLS) determined the CNF and CNC effective diameter, 79 and 190 nm, respectively (Figures S1 and S2). Figure 1, where the upper corners present photos of CNF and CNC water dispersions, shows tapping AFM images for CNFs and CNCs with average sizes of 43 and 18 nm, accordingly. CNFs and CNCs were sedimented on the glass slide surface before measurements. The lengths of CNFs and CNCs were about 500 and 220 nm, respectively. 

### 2.2. Sample Preparation

**Figure 2:**
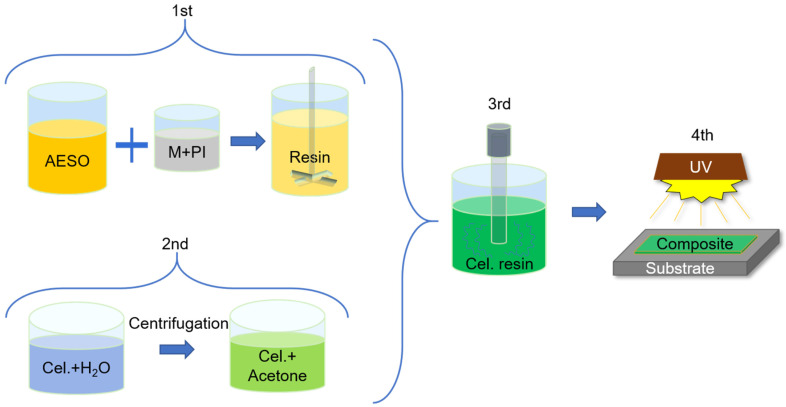
Green nanocomposite preparation scheme: acrylate epoxidized soybean oil (AESO), photoinitiator (PI), TMPTA, HDDA (M), and CNCs and CNFs (Cel.).

The green nanocomposites with nanocellulose contents of 5, 10, 20, and 30 wt% were prepared in a 4-step process, as revealed in Figure 2. The resin preparation was reported by authors elsewhere [15]. Briefly, the neat resin formulation was adjusted in the first preparation step, as shown in the first step of Figure 2, by using the mechanical blending of the oligomer (AESO) with the reactive diluents (TMPTA and HDDA) and photoinitiator (PI). The PI content was 3 wt%. CNCs and CNFs were separated from the water suspension before mixing with the resin. Water in the suspensions was replaced via solvent-assisted centrifugation repeated for 4 cycles using acetone solvent. Nanocellulose in acetone was received and introduced in the resin, as shown in the second and third steps of Figure 2. It involved 1-h ultrasonic dispersion using a Hielscher Ultrasonic Processor UIS250V (Teltow, Germany), simultaneously maintaining cooling within the water bath. The homogeneous composite resin was placed in complete darkness under the fume cupboard to evaporate surplus solvent until a constant weight. In the fourth step, the green nanocomposites films were obtained by UV-curing fabrication. The loaded resins were applied on a glass substrate using an applicator with a thickness of 200–250 µm, and they were then cured under a 5.5 W UV-LED lamp with a wavelength of 405 nm, maintaining a 2.5 cm distance between the light source and the substrate. The nanocellulose particle-loaded resin compositions are represented in Table 1. The bio-based content in the green nanocomposites varied from 63.1 up to 71.4%. The sample is abbreviated as either CNC or CNF with the indication of the wt% content of nanocellulose particles, while neat reference resin is referred to as 0 wt%.

### 2.3. Characterization

The atomic force microscope (AFM) (CP II Scanning Probe Microscope (VEECO, Plainview, NY, USA)) was operated in noncontact mode. The nanocellulose dispersion was dropped on the glass substrate and dried under ambient conditions before measurements.

The UV-VIS spectra of all samples in transmittance mode were measured using a SolidSpec3700 UV-VIS-NIR Shimadzu (Kyoto, Japan) spectrophotometer in the wavelength range of 240–700 nm. The 500 nm spectral line was chosen to compare the transmittance data. UV-cured samples 200–250 µm in thickness were used.

Fourier-transform infrared spectroscopy in attuned total reflectance mode (FTIR-ATR): A Nicolet 6700 (ThermoScietific, Waltham, Germany) was used, the FTIR-ATR resolution was 4 cm^−1^, and the region was 400–4000 cm^−1^, where the average spectrum of sixteen scans of every specimen is shown.

Thermal gravimetry analysis (TGA): A Mettler TG50 instrument (Greifensee, Switzerland) was used to determine the material thermal stability. Measurements were performed for samples with a weight of about 10 mg and a heating rate of 10 °C/min from room temperature up to 750 °C, under a nitrogen (N_2_) atmosphere.

Dynamic mechanical analysis (DMA): A Mettler SDTA861e (Greifensee, Switzerland) dynamic mechanical analyzer (USA) was used for thin-film samples with dimensions of 8.5 × 4 × 0.3 mm, a 1 Hz frequency, a force of 10 N, and an elongation of 10 µm, in the temperature range from −70 to 100 °C and at a heating rate of 3 °C/min.

Scanning electron microscopy (SEM): The structure was analyzed using the Tescan Vega II instrument (Brno, Czech Republic) with a magnification of 1000× and an accelerating voltage of 5 kV. Before the analyses, the samples were coated with gold. 

## 3. Results

**Figure 3:**
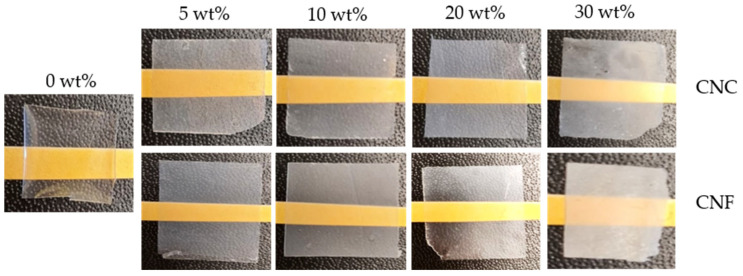
The cured 0 wt% neat resin, and the 5, 10, 20, and 30 wt% CNC and CNF nanocomposites.

Figure 3 shows the optical images of the cured 0, 5, 10, and 20 wt% CNC and CNF nanocomposites. The addition of CNCs and CNFs reduced the translucency of the materials. UV-VIS measurements showed an absolute transmittance of 86% at 500 nm for the cured resin, while both cellulose fillers decreased the transmittance significantly (Supplementary Materials Table S1). The 10 wt% loading decreased the transmittance up to 35 and 73% for CNCs and CNFs accordingly. The obtained nonmonotonous changes in the transmittance values from the filler content could be explained by a mild nanocellulose agglomeration and segregation. Nevertheless, even at a load of 30 wt%, the translucency remained for both fillers to some extent. The 30 wt% CNF nanocomposites had the lowest translucency. 

**Figure 4:**
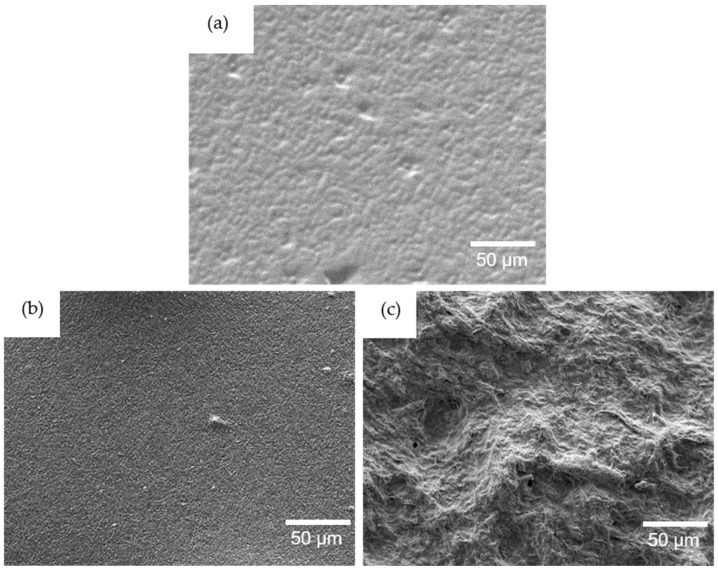
SEM micrographs with 1000× magnification of 0 wt% (**a**) and 10 wt% of CNC (**b**) and CNF (**c**) nanocomposite surfaces.

The 10 wt% nanocomposite structure was characterized by SEM, as seen in Figure 4 at 1000× magnification. The 0 wt% neat resin sample had a relatively smooth surface structure. In comparison, the CNC nanocomposite (Figure 4b) showed an exceptionally homogeneous dispersion that resulted in a nanostructured surface development. Almost no defects could be seen, with only a very few agglomerates revealed. This demonstrates that the AESO-based polymer matrix had excellent interaction and adhesion with CNCs. Otherwise, the CNF nanocomposite (Figure 4c) showed a strongly developed surface structure, which is much rougher when compared to CNCs. The fibers network, i.e., the mesh, was patterned. This indicates that CNFs could create an entangled nanofiber mesh-like structure in the polymer matrix, as demonstrated by Galland et al. for the hyperbranched acrylate matrix with several loadings of nanocellulose nanofibers [34]. 

The UV-curing kinetics of the nanocomposite films were analyzed by FTIR-ATR. Analysis of the curing kinetics of the neat resin has been thoroughly discussed elsewhere [15]. All the characteristic absorption bands are reported in Table 2. The measurements of C=C and C=O bonding absorptions at 810 and 1727 cm^−1^ for 1–10 s of UV-light-cured neat resin showed that an irradiation time of 2.4 s corresponds to an optimally developed polymer chain network for the best combination of crosslinking density and performance characteristics.

**Figure 5:**
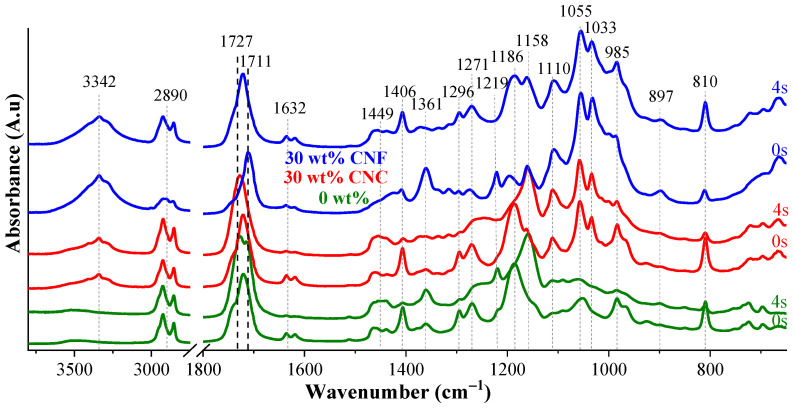
FTIR spectra of uncured and cured 0 and 30 wt% CNC and CNF nanocomposites. The curing time was 4 s.

The spectra of uncured and cured nanocomposites loaded with 30 wt% CNFs and CNCs are represented in Figure 5 alongside spectra of the 0 wt% neat resin as a reference. All characteristic absorption bands of the nanocellulose were assigned from FTIR measurements shown in Figure S3. The broad peak from approximately 3000 to 3650 cm^−1^ is related to –OH stretching vibrations [35,36]; the peak at 2900 cm^−1^ is assigned to the C–H stretching vibration [37]; the peak at 1430 cm^−1^ represents CH_2_ symmetric bending [37], while the peak at 1316 cm^−1^ is assigned to CH_2_ wagging, and C–OH in-plane bending at C6 can be observed at 1204 cm^−1^ [35]. The band at 1160 cm^−1^ corresponds to C–O–C asymmetric stretching at the β-glycosidic linkage [35]. Other characteristic absorption peaks of C–O in cellulose can be observed approximately at 1055 cm^−1^ [36,38] and at 1028 cm^−1^, and C–O-specific C6 stretching is represented by the band at 985 cm^−1^. The signature peak at 897 cm^−1^ is assigned to C–O–C asymmetric stretching at β-glycosidic linkages of amorphous cellulose [35,39]. 

The peak of the C–O bond at C6 stretching belonging to cellulose shifted from 1028 to 1033 cm^−1^, while similarly, a shift in the second peak was observed from 1203 to 1219 cm^−1^ (Figure 5). These peaks were not observed for the neat resin, and they contributed to the developed cellulose–polymer interaction. Meanwhile, absorption bands attributed to nanocellulose at 1316 and 1430 cm^−1^ representing CH_2_ wagging and C–H stretching vibrations could not be observed, due to the overlapping. Nonetheless, the separate peak applicable only to the nanocellulose fillers at 897 cm^−1^ related to C–O–C asymmetric stretching remained clear [39]. 

**Figure 6:**
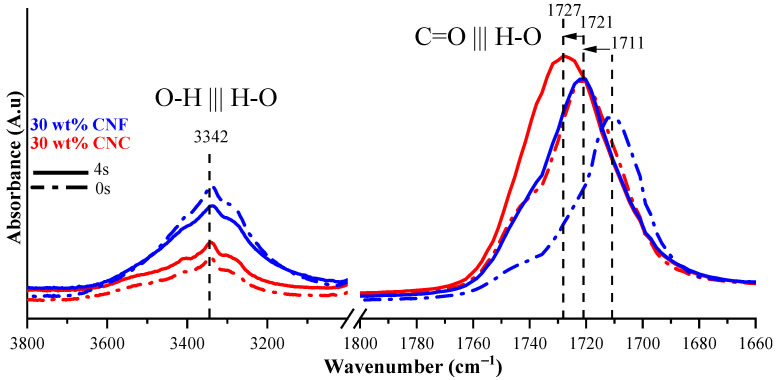
FTIR spectra of uncured (**dashed lines**) and cured (**solid lines**) 30 wt% CNC and CNF nanocomposites.

The –OH stretching vibration of the nanocellulose was revealed at 3342 cm^−1^ (see Figure 6). The absorption was higher for CNF nanocomposites produced by their more elevated surface. Lui et al. suggested the current absorption band for H-bonding assessment between the hydroxyl groups of nanocellulose nanofibers [39]. The C=O absorption band at 1727 cm^−1^ of polymeric chains corresponds directly to the developed H-bonding (C=O ||| H–O) between the polymer matrix and nanocellulose nanofibers; then, H-bonding (H–O ||| H–O) between nanocellulose nanofibers was revealed at 3342 cm^−1^ in Figure 6. Ratios between C=O and O–H peak intensities (I_1727_/I_3342_ and I_1721_/I_3342_) for 30 wt% CNC and CNF composites were 4.4 and 2.4, respectively. The almost 2-fold higher absorption intensity ratio for the CNCs reflects the higher nanocellulose interaction with the polymer matrix than the CNFs. Indeed, as shown in Figure 6, the C=O absorption band of the CNC nanocomposites shifted to longer wavenumber values, while the H–O absorption intensity strongly increased for nanocomposites compared to the uncured resins. Liu et al. attributed absorption bands shifts to the developed H-bonding interactions at the interface between cellulose particles and polymer matrix [39], which was more efficiently revealed for the CNC than the CNF nanocomposites. Attributed absorption bands shifted to the developed H-bonding interactions at the interface between the cellulose particles and polymer matrix [39], which were more efficiently revealed for CNC than for CNF nanocomposites. 

**Figure 7:**
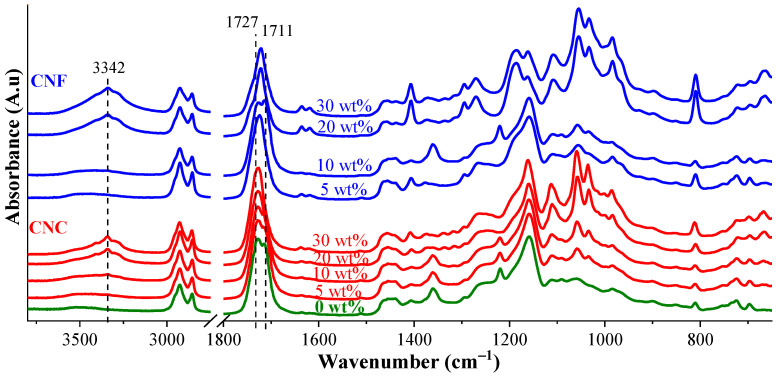
FTIR spectra of cured 0 wt%, and 5, 10, 20, and 30 wt% CNC and CNF nanocomposites. The curing time was 4 s.

The absorption bands at 810 and 1727 cm^−1^ associated with C=C and C=O have been assessed for the curing performance of resin compositions. An extensive catalog of FTIR spectra with changes in peak intensities during irradiation for curing times of 0, 4, 6, 8, and 10 s for all obtained composites can be seen in Supplementary Materials (Figures S4–S11) and optimal curing times for all resins can be seen in Figure S12. The characteristic spectra of the cured 0, 5, 10, 20, and 30 wt% CNC and CNF nanocomposites can be seen in Figure 7. 

**Figure 8:**
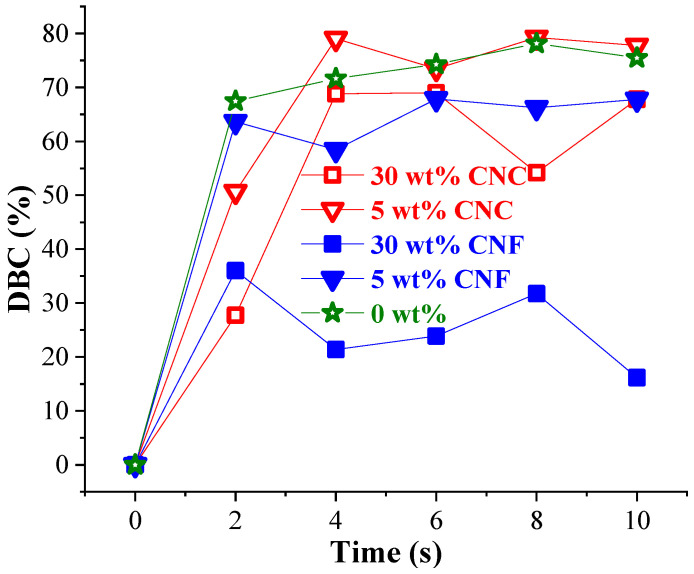
Double bond conversion for 0 wt%, and 5 and 30 wt% CNC and CNF nanocomposites.

The CNF nanocomposites’ absorption bands, although in the same wavenumber ranges, were much more intensive compared to the CNC nanocomposites. The higher content of nanocellulose contributed to the overall decrease in polymer resin characteristic absorption intensities. A similar explanation was reported by Yang et al. [40], who noted absorption intensity changes related to the development of the hydrogen bonding crosslinks between monomer’s OH, C=O, and O=C–O groups and cellulose’s –OH groups. The calculated double bond conversion (DBC%) [15,41] reveals a similar curing trend for the composites, as shown in Figure 8. Figure S13 provides additional information about the nanocomposites’ DBC%. 

The steepest curing and the highest DBC% were received for the neat resin. After 2 s of irradiation, 67% of the double bonds were converted, followed by the highest 78% DBC% reached after 8 s. Herein, achieved curing time characteristics were considerably enhanced compared to the curing of poly(methyl methacrylate) and 1,6-hexanediol dimethacrylate formulations discussed by Zhang et al. [42], where after 5, 10, and 15 s of UV-light irradiation, the DBC% reached 18, 55, and 72%, respectively. Steyrer et al. showed that the additional post-curing at elevating temperatures of the UV-light irradiation had increased DBC% by 2-fold [41]. Nevertheless, we report that we have reached a 1.35-fold higher DBC% than Steyrer et al. did without the additional post-curing at elevated temperatures. The CNC nanocomposites’ curing with 70% and 80% of DBC% took place in the first 4 s indicated for 30 and 10 wt% loadings, respectively. The CNFs had a more substantial impact on UV-light curing than the CNCs did. A lower degree of DBC% was achieved for the CNF nanocomposites compared to the CNCs. The curing process seemed to reach equilibrium after 2 s of irradiation for the CNF composites, where 36 and 41% of DBC% correspond to 30 and 10 wt% loadings, respectively. In addition, 72% of DBC% was received after 4 s of curing for the 30 wt% CNC sample. Other CNC and CNF samples showed a similar trend (Figure S13). 

The UV-light curing process impacts the developed macromolecular chain network, which was revealed by the crosslinking density N and the molecular weight between crosslinks M_c_ that have been calculated according to the Flory–Rehner equation [43] and corresponds to the empirical approach used by authors before [44]. Table 3 presents the calculated polymer chain network N and M_c_ values for prepared UV-cured compositions. The UV-cured nanocomposite containing 30 wt% of CNFs and CNCs is characterized by the 2-fold and 6-fold-enhanced M_c_ compared to the 0 wt% sample.

**Figure 9:**
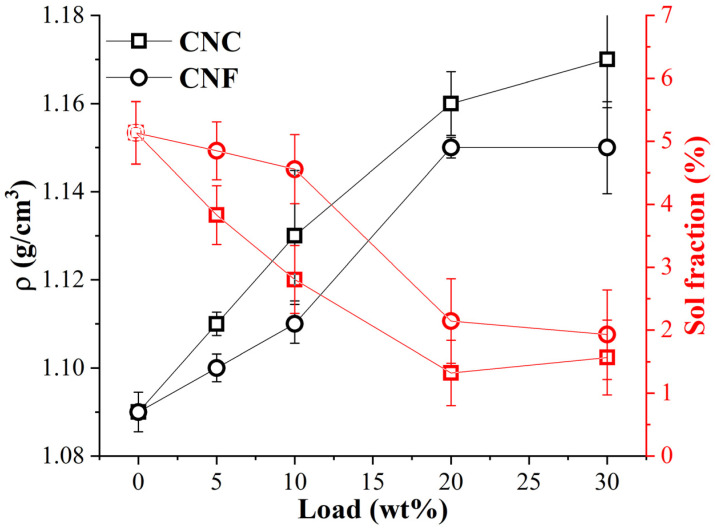
Density and sol fraction dependence on the CNC and CNF content.

The density and gel fraction of the UV-light-cured nanocomposites revealed similar observations, as shown in Figure 9. It indicates that by increasing the content of nanocellulose in the nanocomposites, the density rose by almost 6 and 9% for the CNFs and CNCs, accordingly. The CNCs and CNFs had the same absolute density value of around 1.6 g/cm^3^ [8,23,45]. The experimental density of CNC materials was higher than that of CNFs, due to the denser stacking of crystalline short rod-like CNCs than the entangled CNF nanofibers [23]. Sol fraction, i.e., dissolved polymer fraction [46], was also acquired in Figure 9, which complemented curing efficiency. The observed incremental decrease in sol fraction from 5 to 2% for the neat resin and 30 wt% nanocomposites, correspondingly, relates to the observed DBC% remarkable drop after nanocellulose incorporation into the polymer matrix (Figure 8).

**Figure 10:**
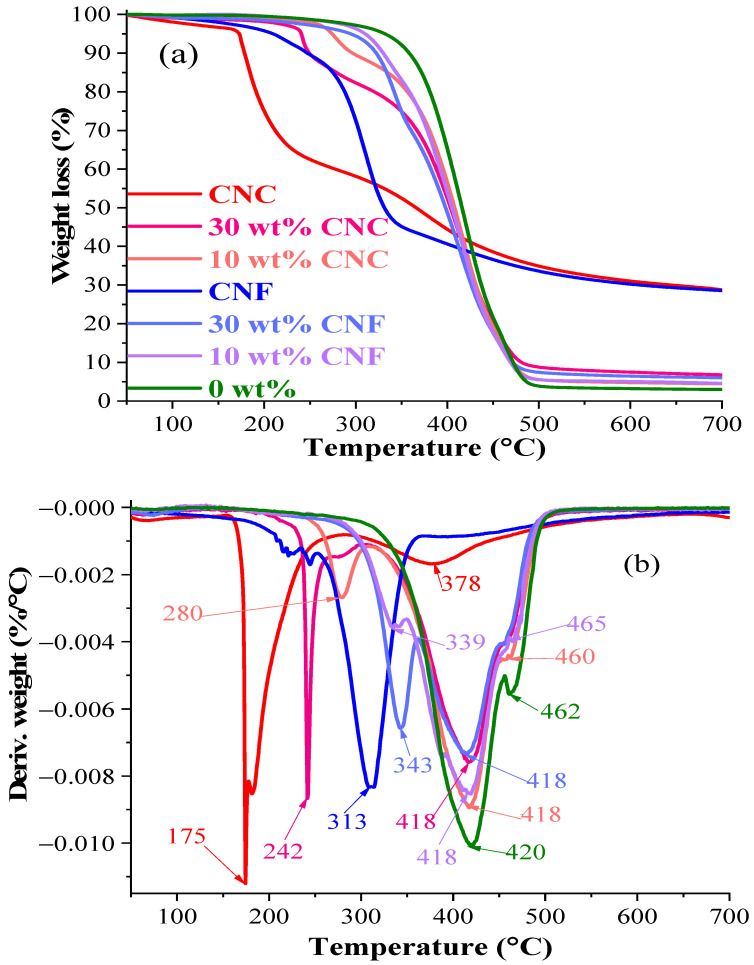
Thermal stability of 0 wt%, and 10 and 30 wt% CNC and CNF nanocomposites: TG weight loss (**a**) and DTG derivative weight (**b**).

Thermogravimetric analysis (TGA) was used to determine the thermal stability of the nanocomposites expressed as weight loss during the uniform heating rate of 10 °C/min in an inert nitrogen atmosphere. The weight loss curves and derivative curves of the neat resin and nanocomposites are shown in Figure 10 and Figure S14. The cellulose composites are known for lower thermal stability than the neat polymer materials [47]. It was identified before that reactive diluent increases the thermal stability, and the thermal degradation maxima at 462 °C are attributable to reactive diluents [13]. CNF samples, by themselves, have a higher thermal stability than CNC samples do, which is explained by the higher CNC surface area that provides larger exposure to the heat [48]. An enhanced nanoparticle–matrix interaction, observed with CNC nanocomposites via FTIR analysis, ensures better thermal protection by the polymer matrix [49]. In addition, 5% weight losses of the 0 wt% and 30 wt% CNFs and CNCs were observed at 332, 294, and 240 °C (Figure 10), correspondingly. The determined temperature at maximal degradation (T_max_) was 313 and 175 °C, respectively, for CNFs and CNCs. However, it should be mentioned that above 320 °C, the thermal stability seemed to be higher for the CNCs than for the CNFs, as observed in Figure 10a. 

Three thermal degradation peaks for nanocomposites can be distinguished in Figure 10b. The first degradation step is attributed to a nanocellulose close to the samples’ surface as the polymer matrix usually provides some thermal protection to the natural fibers [49]. The first step of weight loss for nanocomposites is limited to weight loss from 0 to 20%. The second step relates to the degradation of the main polymer matrix component, as identified elsewhere [13]; the third stage of degradation attributes to the reactive diluents component contribution. Figure S14 showed decreased thermal stability for the CNF and CNC nanocomposites, while the CNF samples thermally degraded at higher absolute temperatures than the CNC nanocomposites did. It was observed that when the nanocellulose content increased, the CNC and CNF nanocomposite thermal stability gradually decreased. We found that the overall thermal stability, if measured by T_max_, did not suffer much compared to the poly(methyl methacrylate)/CNC nanocomposites reported in research by Sain et al., where T_max_ dropped by 9 °C [50]. The tendency of the decrease in thermal stability, followed by increasing particle content, is compiled in Table 4. The 0 wt% sample had T_max_ = 420 °C, and the CNF and CNC nanocomposites then showed a 2–5 °C decrease in T_max_, which depends on the nanocellulose and its content. The highest T_max_ drop down to 415 °C was observed for the 20 wt% CNC, the 30 wt% CNF, and the CNC nanocomposites. T_max_ dropped only by 1.2%, but the first 10% of weight loss was reached at 30.4% and 10.0% lower temperatures for 30 wt% CNC and CNF nanocomposites, respectively, as can be seen in Figure 10b. As for char yields, nanocellulose increased the leftover char yield, but it seems that neither the CNCs nor the CNFs mattered. Both nanocelluloses char yields at 700 °C were around 30%. 

The incorporation of nanocellulose can significantly increase the mechanical properties as interactions in the nanoscale directly impact the polymer interphase formation. Nanocomposites provide a different response to DMA continuous cyclic load depending on the nanocellulose content, dispersion degree, and formed interface adhesion between the reinforcement and polymer matrix. At lower nanocellulose loadings, the CNFs would act as separate reinforcement nanofibers, but at higher loadings, the formation of a continuous entangled nanofiber mesh-like network has been reported [23]. The CNCs were observed as short rod-like nanoparticles (Figure 1); therefore, they were homogeneously dispersed in the polymer (Figure 4), while the good adhesion with the matrix remained. 

**Figure 11:**
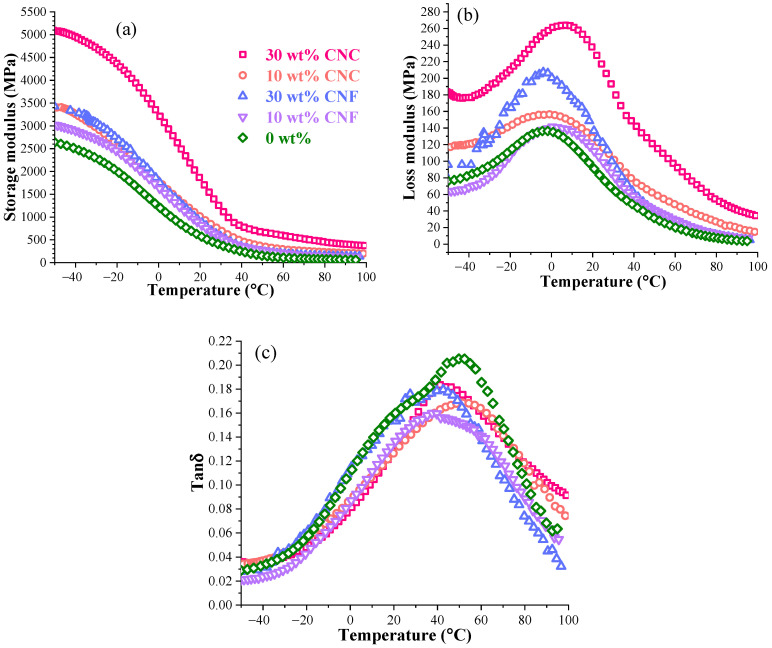
Storage modulus (**a**), loss modulus (**b**), and loss factor tan δ (**c**) of 0 wt%, and 10 and 30 wt% of the CNC and CNF nanocomposites.

The DMA measurement results of the CNF and CNC nanocomposites are illustrated in Figure 11 and Figure S15, respectively. The glass transition temperature and storage modulus values at different temperatures are summarized in Table 5. The 5 wt% loadings of CNFs and CNCs showed a medium increase in the storage modulus values (Figure S15). A significant storage and loss modulus increase was achieved for the nanocomposites with a nanocellulose load of 10–30 wt%, where a good reinforcement network was established, as indicated by the increased absolute values in both glassy and viscoelastic states. 

This presents an opportunity for optimizations of the properties as the formation of such a network yields other benefits such as improved barrier properties [51,52]. The shape differences of the nanocellulose are further expressed in the viscoelastic state, where CNCs show significantly higher values than the CNFs do. A 3.3-fold and 2.0-fold increase in the storage modulus at 80 °C was observed for 10 wt% of the CNC and CNF nanocomposites, correspondingly (Table 5). However, the 30 wt% loading of the nanocellulose produced a remarkable increase in the storage modulus at −50 °C—1.9-fold and 1.3-fold, and at +80 °C—6.2-fold and 2.1-fold, for the CNCs and CNFs, accordingly. Wool et al. reported [53] reinforcement acrylated epoxidized soybean oil with keratin fibers cured with a cumyl peroxide free-radical initiator. The modulus reached 2800 MPa at 0 °C with 30 wt% of the nanocellulose. Herein, higher performance was achieved for the 15 wt% CNC loading.

A loss modulus increase was observed for highly loaded nanocomposites compared to the neat polymer. In addition, 20–30 wt% loadings of the CNCs and CNFs significantly affect and restrict polymer segment motions; thus, the phase transition requires more energy, and subsequent slippage between the particles and matrix results in higher dissipated energy as heat [54]. If CNCs and CNFs are compared, then CNCs present a gradual increase due to the particle nature, size, and geometry, but CNFs do not follow the same trend. Polymer chain adsorption on CNF nanofibers is more restricted due to their morphology and entanglement [23]. CNFs tend to agglomerate due to strongly developed hydrogen bonding between entangled nanocellulose nanofibers, as indicated by FTIR spectra.

The CNC and CNF effects on the damping properties are observed in Figure 11c. Tan delta showed lower peak values for all nanocomposites than the 0 wt%. In this case, all samples followed a similar trend in line with the expected elastic response of the composite promoted by the addition of a rigid nanocellulose reinforcement, as reported by other authors [48]. The 0 wt% neat resin showed two glass transitions at 40 and 50 °C, as reported elsewhere [15]. The nanocellulose significantly affected the formation of the cured polymer chain network, which results in a higher rigidity and lower tan delta peak values for the CNC and CNF nanocomposites. The lowest tan delta values were observed for the 10 wt% compositions with values of 0.17 for the CNCs and 0.16 for the CNFs. The 5–30 wt% CNCs shifted the glass transition to a higher-temperature region, about 10–20 °C. The glass transition temperature, obtained from the tan delta peak maximum value, did not fully reflect the peak shifting trend; it could be explained by a relatively broad peak due to the formation of the crosslinked chain networks [55]. The tan delta peak shifts by 5–15 °C indicate significantly stronger interactions between the CNCs and the polymer matrix than the CNFs. These results coincide with the literature, where CNC addition to the thermoset matrix strongly increases the glass transition temperature [56].

DMA characteristics of the glassy ($E_{g}^{\prime }$ at −45 °C) and viscoelastic ($E_{v}^{\prime }$ at 95 °C) storage modulus values listed in Table 5 were used to analyze the nanocellulose impact on the polymer matrix. The observed increased $E_{g}^{\prime }$ correlates with the degree of entanglement and particle dispersion efficiency, and $E_{v}^{\prime }$ correlates with the crosslinking and interaction between the particles and the polymer matrix. Parameter *C* calculated from Equation (1) describes the probability of the composite to enter its glass transition region faster or, in other words, a relative measurement of the modulus drop while increasing temperature and the material passing *T_g_* [57,58]:

(1) $$C=\frac{{\left(E_{g}^{\prime }/E_{v}^{\prime }\right)}_{composite}}{{\left(E_{g}^{\prime }/E_{v}^{\prime }\right)}_{matrix}}$$

**Figure 12:**
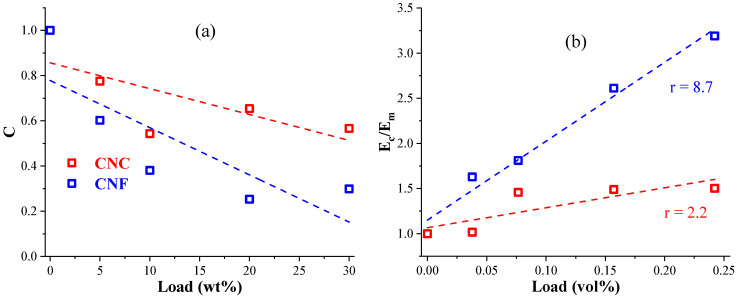
The C factor as a function of weight content (**a**) and the glassy storage moduli ratio (**b**) according to Equation (2) for 0 wt%/CNC or CNF wt% as a function of volume content of the CNC or CNF nanocomposites.

The maximum stress transfer between nanocellulose and the polymer matrix is shown for factor *C* below 1.0. Effectively dispersed particles that are compatible with the matrix, and a lower value of *C* indicates reinforcement effectiveness [58]. All prepared nanocomposite values were in the range of 0.2 < *C* < 0.8, as seen in Figure 12a. This further demonstrates that the CNC is a more suitable nanocellulose for the AESO-based polymer matrix as a higher *C* factor indicates a proper nanocellulose–polymer matrix attraction and evasion of the agglomeration and restacking. This also shows that the CNF reached the highest *C* at 10 wt% content, while the CNCs showed the highest value at 20 wt%. In addition, a supportive parameter can be used for characterizing particle interaction with the polymer matrix, known as a reinforcement efficiency factor—*r* [59]. The nanocomposite’s storage modulus (*E_c_*) and the polymer matrix’s storage modulus (*E_m_*) values are related by an empirical relationship, which can be written using Einstein’s considerations for suspensions with rigid particles [57,60]:

(2) $$E_{c}=E_{m}(1+rV_{f})$$

where *V_f_*—the volume fraction of a particle in the composite.

Following Equation (2), the ratio *E_c_/E_m_* was calculated and plotted against nanocellulose volume % as the graph’s slope in Figure 12b provides *r* values. Calculations for *E_c_/E_m_* were performed using the glassy storage modulus. Linear trends are shown with dotted lines. As expected, *r* for CNCs showed a higher value than that for CNF nanocomposites, 8.7 and 2.2, respectively. Dispersion issues resulting in agglomerates and imperfect bonding or a reduced contact surface between nanoparticles and the polymer influence the nanocomposite’s storage modulus values [59]. CNFs, similarly to previous observations, offered limited performance at contents above 10 wt%, as indicated by the *r* factor that had almost identical values for 10 to 30 wt% nanocomposites (1.45 and 1.5, respectively). This demonstrates that the positive effect on the mechanical properties is limited by the content of CNFs and reaches optimal loading at 10 wt%. Remarkably, CNCs demonstrated a gradual increase with *E_c_/E_m_* even above 20 wt% loadings, reaching 3.19 for 30 wt%. This shows how the morphology of the nanoparticles has a direct effect on the thermoset polymer matrix.

## 4. Conclusions

The present study contributes to the understanding of the nanocellulose reinforcing efficiency in prepared UV-curable vegetable oil-based thermoset polymer nanocomposites. Cellulose nanocrystals (CNCs) and cellulose nanofibers (CNFs) of 5–30 wt% were introduced in acrylated epoxidized soybean oil-based resin. We compared the nanocellulose reinforcing effect on polymer resin curing, and on the thermal, thermomechanical performance, and structural properties. FTIR data showed that nanocellulose incorporation significantly enhanced the celluloses’ hydroxy-group absorption intensity and shifted the polymer carbonyl-group absorption band to a longer-wavenumber region. A more pronounced effect was revealed for the CNC samples related to the hydrogen bonding development between the polymer chains and cellulose. The CNF filler formed agglomerates of mesh-like network structures in the nanocomposites, evidenced by SEM analysis. Thermal stability analysis shows that the CNC nanocellulose affected the material thermal degradation significantly at 10 wt% loadings. The incorporation of CNFs proved to be preferable over CNCs for the material’s thermal stability because the 30 wt% CNF nanocomposite thermal degradation was 20 °C higher than those of the CNC samples. DMA measurements for the 10 and 30 wt% CNC nanocomposites demonstrated superior stiffness performance compared to the CNF samples by remarkably increasing the storage and loss modulus values. The storage modulus was 3-fold and 6-fold at 30 °C and 80 °C for the 30 wt% CNC nanocomposites, respectively, while the 10 and 30 wt% CNF samples showed an increase of 1.5-fold and 2-fold, correspondingly. In-depth analysis, using the relative modulus drop by increasing the temperature factor—*C* and reinforcement efficiency factor—*r*, indicated that CNFs reached the highest reinforcement at 10 wt%, but CNCs reached it at 30 wt% nanocomposite loadings. Compared to the CNF samples, CNC nanocomposites showed superior exploitation properties with high filler loadings. 

This study aimed to explore the relevant reinforcing issues associated with the use of cellulose nanocrystals and nanofibers to produce advanced renewable composites for coatings, adhesives, and additive manufacturing applications. The present research is to be further expanded to UV-light-curable coatings and stereolithography 3D printing applications of the proposed renewable resin compositions.

## Figures and Tables

**Table 1:** Obtained green nanocomposite compositions.

Load, wt%	Biobased Content, wt%
0	63.1
5	64.8
10	66.4
20	69.1
30	71.4

**Table 2:** FTIR absorption peaks of the neat resin.

Absorption Peak cm^−1^	Functional Group
810	C=C out-of-plane bending
985	CH_2_=CH–R asymmetric band
1055	C–H asymmetric stretching
1158	C–O–C stretching vibrations of ester
1186	C–O–C stretching vibrations
1271	O=C–O stretching vibrations of ester
1406	CH_2_=CH scissoring band for terminal alkene
1449	CH scissoring band in –CH_2_–
1632	CH_2_=CH
1727	C=O stretching vibrations
2890	–CH_2_–, –CH_3_ groups C–H stretching

**Table 3:** Crosslink density and molecular weight between crosslinks.

Load, wt%	M_c_, g/mol		N, 10^3^ mol/cm^3^	
	CNF	CNC	CNF	CNC
0	141		8.2	
5	111	89	10.0	12.6
10	69	43	16.0	26.1
20	76	29	15.2	40.0
30	69	23	16.8	51.1

**Table 4:** Weight loss at thermal degradation.

KERRYPNX	Load, wt%	T °C When Weight Loss						T_max_, °C	Char, wt%
		5%	10%	30%	50%	70%	90%		
Neat resin	0	332	358	395	416	437	472	420	3
CNF		208	247	302	332	606	-	313	29
CNF	5	314	349	391	414	436	473	418	4
	10	310	296	380	408	431	471	418	5
	20	297	275	365	399	426	467	418	7
	30	294	249	367	403	431	481	415	8
CNC		173	178	212	366	644	-	175	29
CNC	5	317	336	383	407	430	469	418	4
	10	274	330	378	405	429	469	418	5
	20	264	325	366	401	429	478	415	6
	30	240	322	360	398	426	472	415	8

**Table 5:** Storage modulus at different temperatures and T_g_ of the 0 wt%, and CNF and CNC nanocomposites.

	Load, wt%	Storage Modulus, MPa							T_g_, °C
		−50 °C	−45 °C	−20 °C	0 °C	30 °C	80 °C	95 °C	
Neat resin	0	2640	2578	2007	1219	381	72	59	40/50
CNF	5	2725	2643	2112	1420	371	88	78	40
	10	3022	2967	2441	1627	541	141	125	41
	20	3611	3513	3110	1941	529	134	123	38
	30	3431	3366	2728	1776	565	148	136	37
CNC	5	2704	2630	2121	1531	617	111	100	46
	10	3403	3377	2595	1759	692	230	203	51
	20	3610	3528	2812	2173	1036	352	319	55
	30	5095	5040	4368	3237	1168	450	386	43

## Data Availability

The data that support the findings of this study are available from the corresponding author upon reasonable request. Research is still ongoing.

## Supplementary Materials

The following are available online at https://www.mdpi.com/article/10.3390/nano11071791/s1, Figures S1–S15 and Table S1.
