# Supplementary material for: Cellulose Nanocrystals vs. Cellulose Nanofibers: A Comparative Study of Reinforcing Effects in UV-Cured Vegetable Oil Nanocomposites
Source: Nanomaterials (Basel). 2021 Jul 9;11(7):1791. doi: 10.3390/nano11071791 (PMC8308285; doi:10.3390/nano11071791)
Supplement: Supplementary file 1 [file nanomaterials-11-01791-s001.zip › nanomaterials-1267533-supplementary.pdf]

nanomaterials

Supplementary Materials

Cellulose Nanocrystals vs. Cellulose Nanofibers: A Comparative Study of Reinforcing Effects in UV-Cured Vegetable Oil Nanocomposites

Anda Barkane, Edgars Kampe, Oskars Platnieks and Sergejs Gaidukovs *

Institute of Polymer Materials, Faculty of Materials Science and Applied Chemistry, Riga Technical University, P. Valdena 3/7, LV-1048 Riga, Latvia; Anda.Barkane@rtu.lv (A.B.); Edgars.Kampe@rtu.lv (E.K.); Oskars.Platnieks_1@rtu.lv (O.P.)
* Correspondence: Sergejs.Gaidukovs@rtu.lv

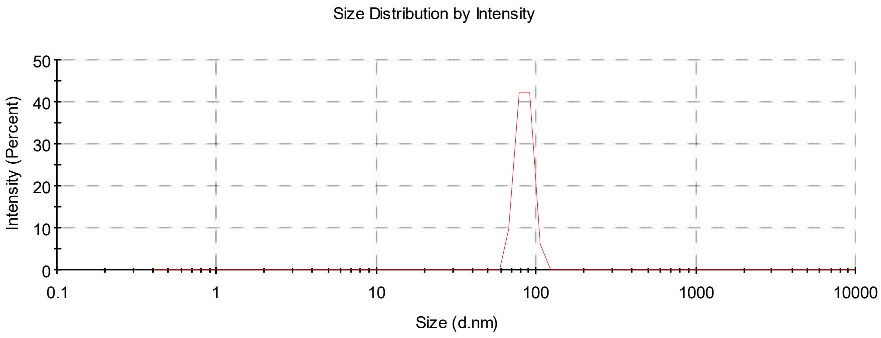

Figure S1. Particle size for CNC.

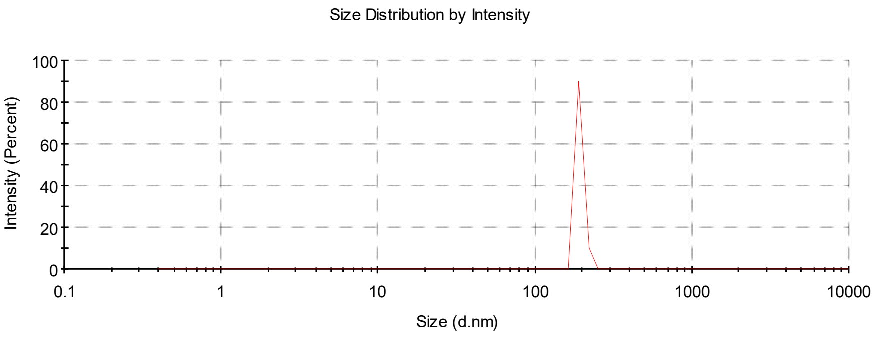

Figure S2. Particle size for CNF.

Table S1. UV-VIS transmittance % at 500 nm.

Load, wt%	Transmittance, %	
	CNC	CNF
0	86	86
5	69	84
10	35	73
20	74	68
30	88	62

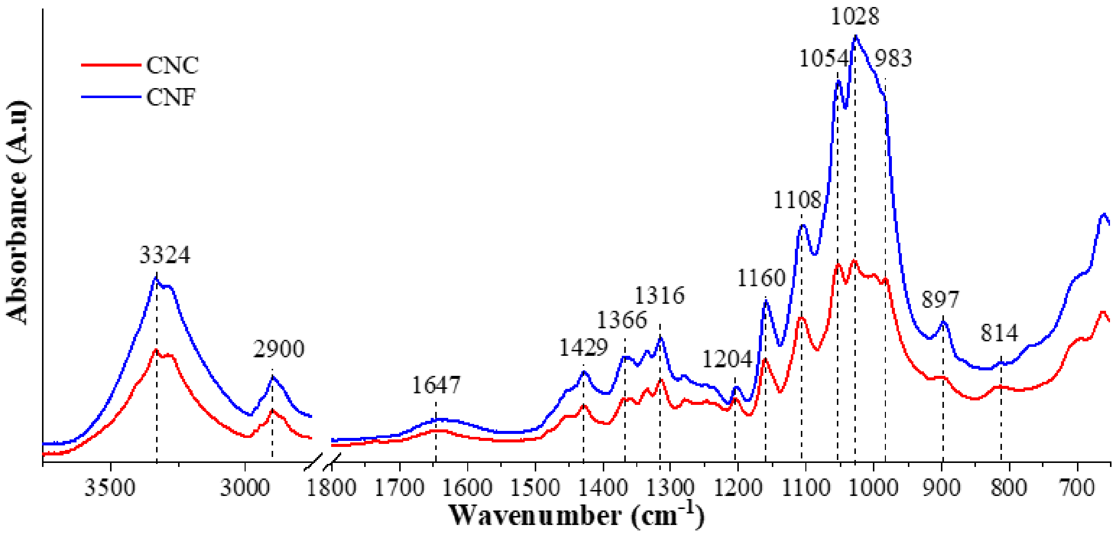

Figure S3. FTIR spectra of CNC and CNF.

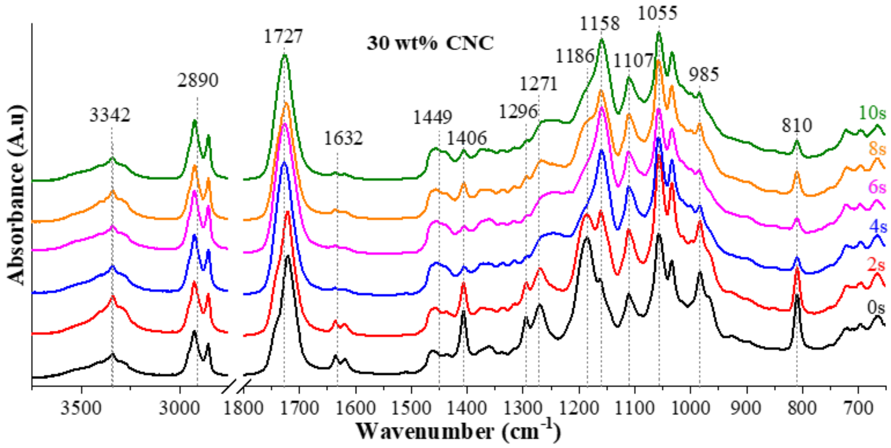

Figure S4. FTIR depending on time for the neat sample loaded with 30 wt% of CNC.

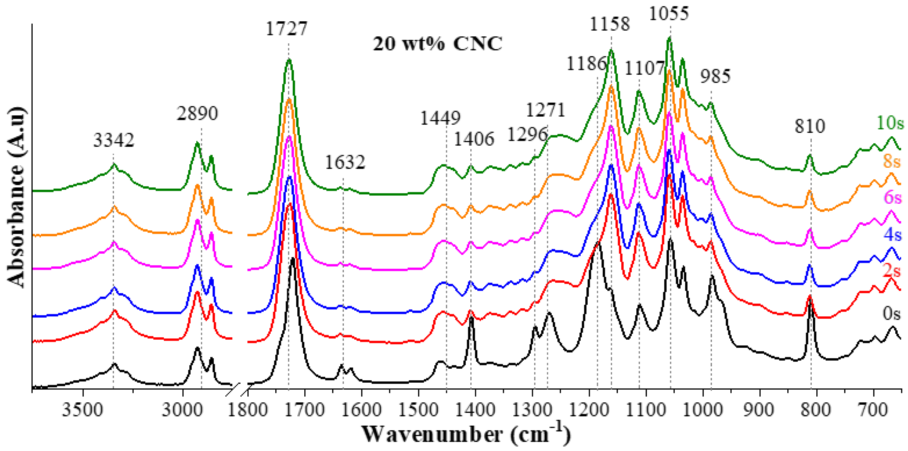

Figure S5. FTIR depending on time for the neat sample loaded with 20 wt% of CNC.

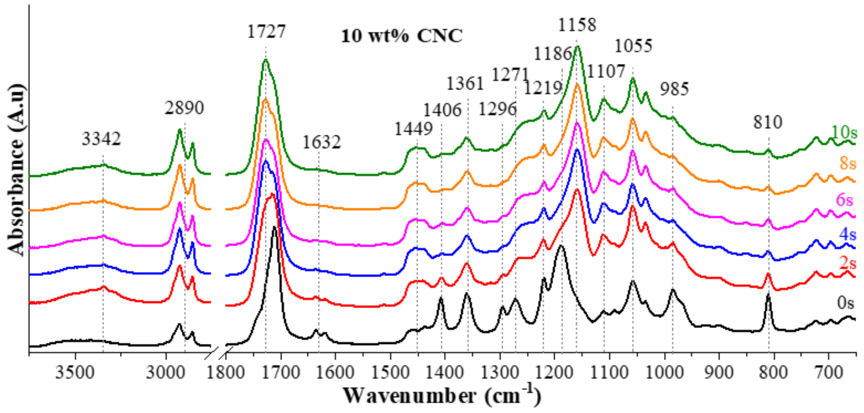

Figure S6. FTIR depending on time for the neat sample loaded with 10 wt% of CNC.

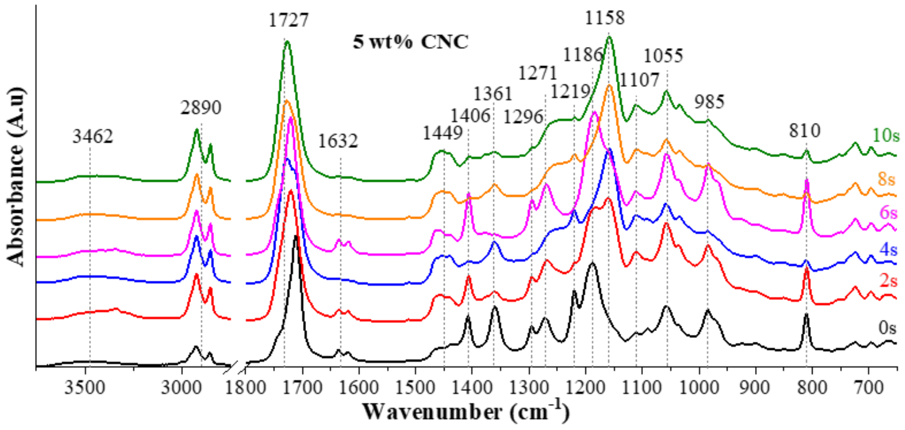

Figure S7. FTIR depending on time for the neat sample loaded with 5 wt% of CNC.

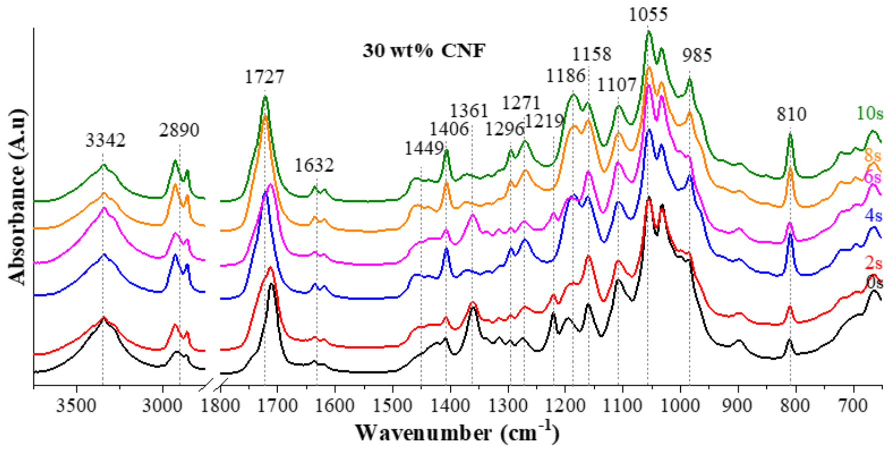

Figure S8. FTIR depending on time for the neat sample loaded with 30 wt% of CNF.

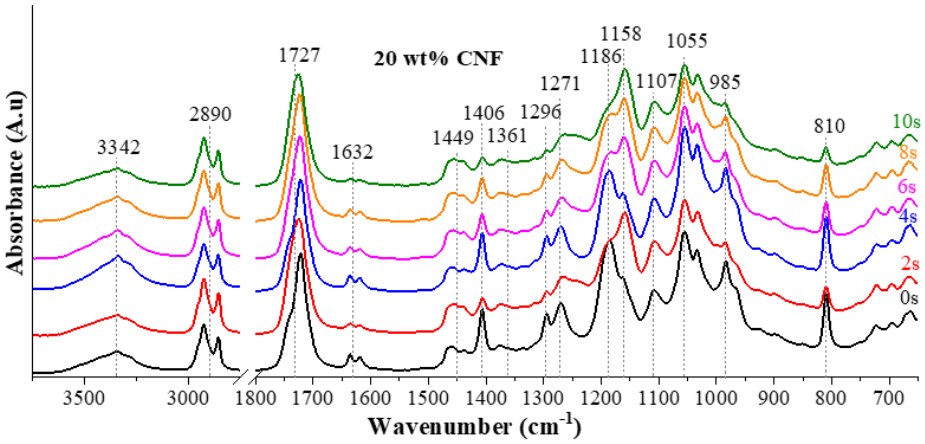

Figure S9. FTIR depending on time for the neat sample loaded with 20 wt% of CNF.

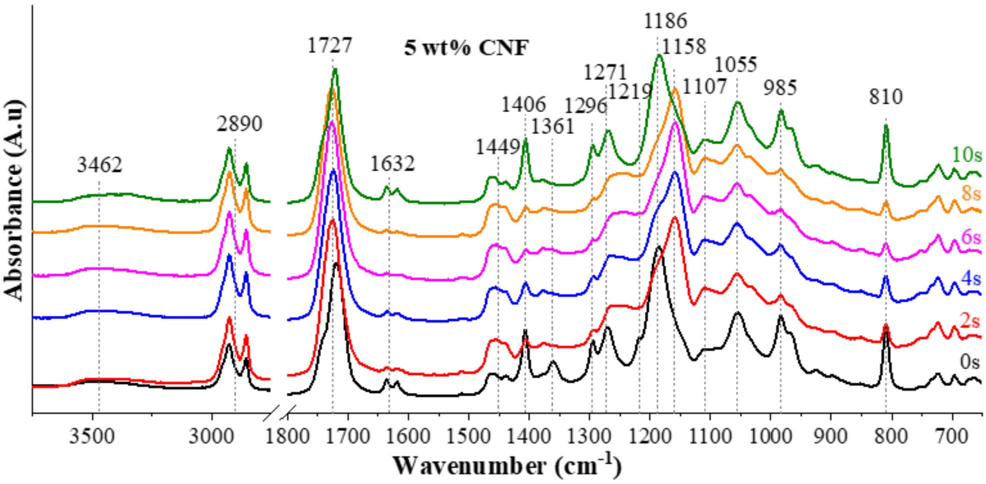

Figure S10. FTIR depending on time for the neat sample loaded with 10 wt% of CNF.

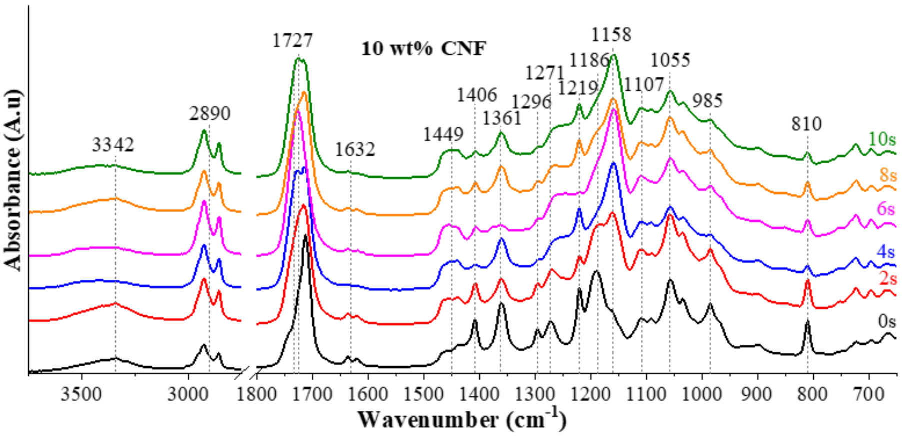

Figure S11. FTIR depending on time for the neat sample loaded with 5 wt% of CNF.

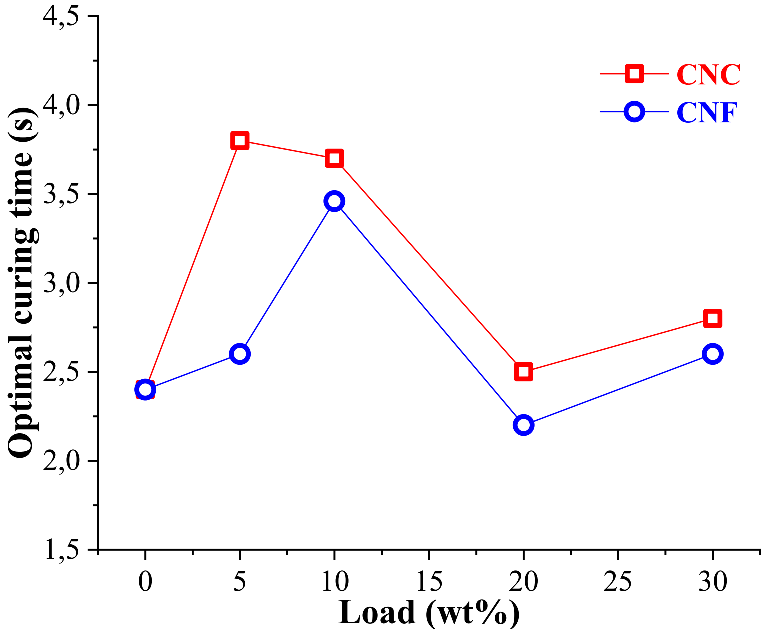

Figure S12. Optimal curing time depending on filler content.

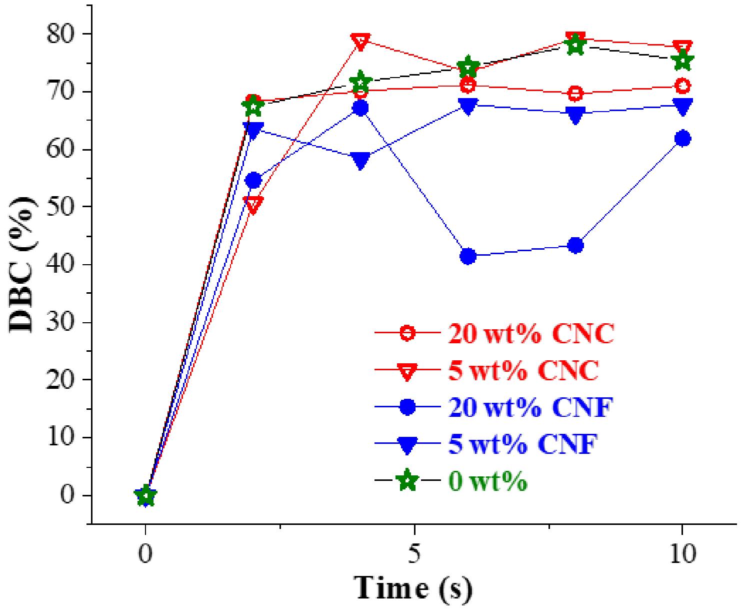

Figure S13. Double bond conversion (DBC%) rate for 0 wt%, and 10 and 20 wt% loaded nanocomposites depending on irradiation time.

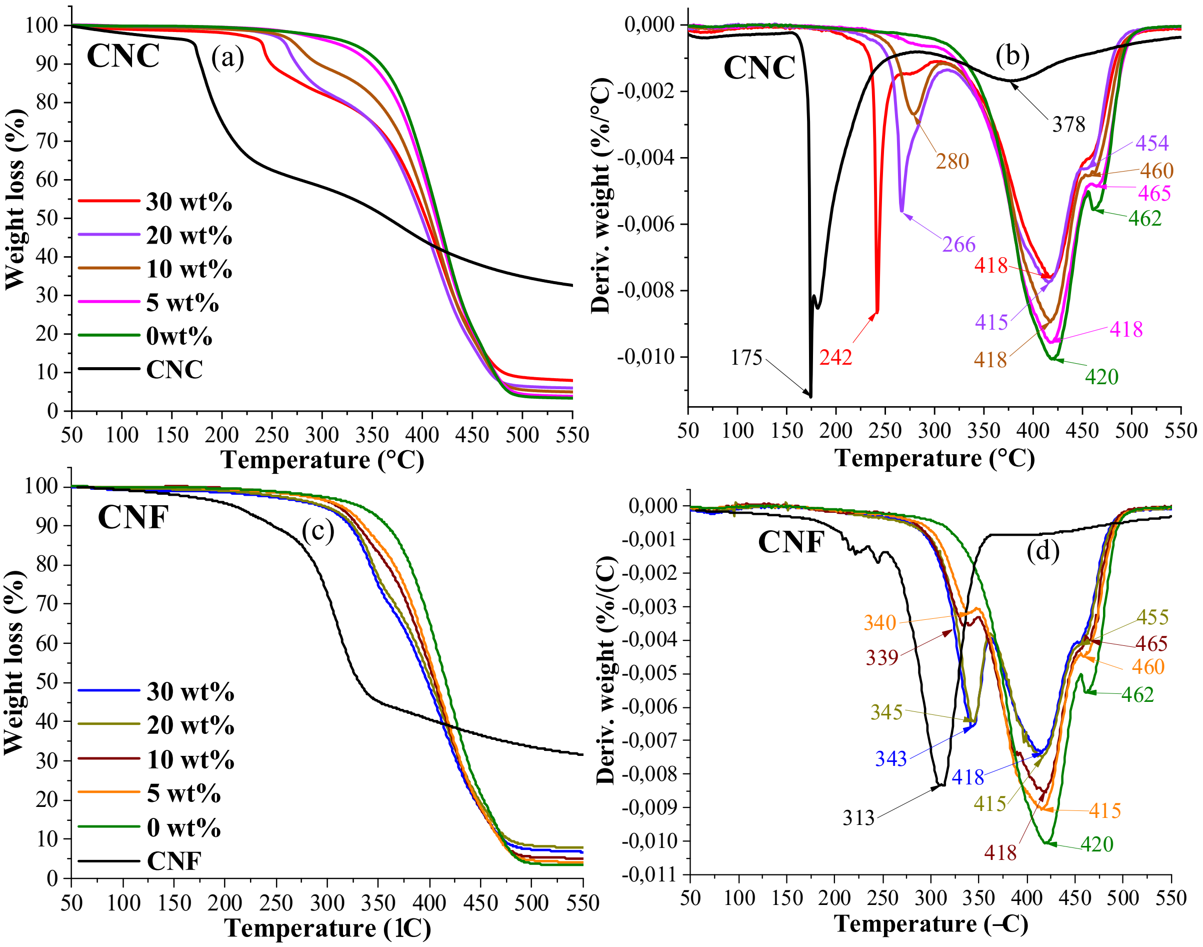

Figure S14. Thermal stability of CNC and CNF-loaded nanocomposites: TG weight loss (**a**),(**c**), and DTG derivative (**b**),(**d**).

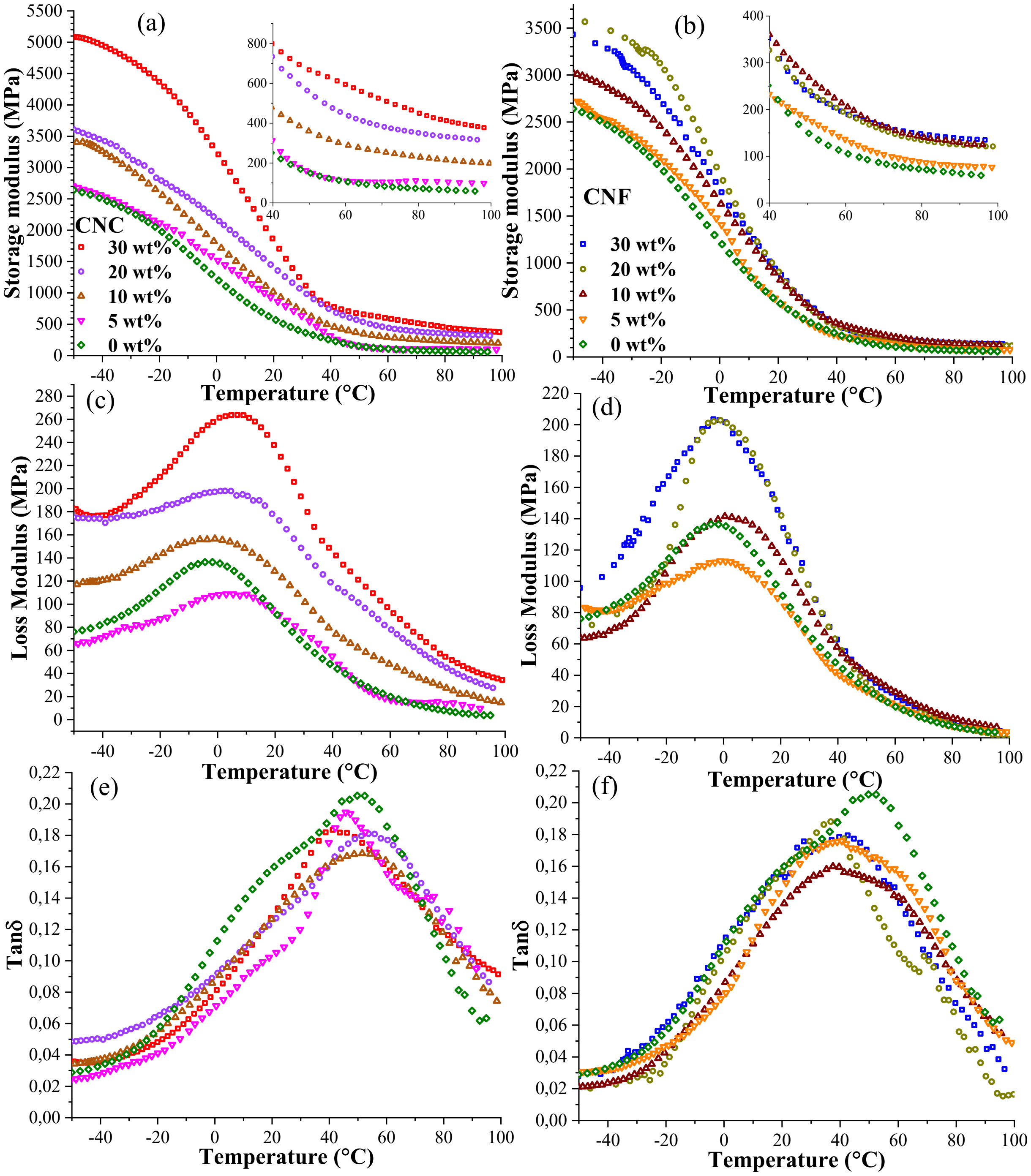

Figure S15. Loss modulus and storage modulus curves of 0 wt%, and CNC (**a**) and CNC (**b**); loss modulus curves of 0 wt%, and CNC (**c**) and CNF (**d**); and loss factor tanδ of 0 wt%, and CNC (**e**) and CNF (**f**).
